# Duplicated Inferior Vena Cava Thrombosis Mimicking Acute Pancreatitis in a COVID-19 Patient

**DOI:** 10.7759/cureus.33220

**Published:** 2023-01-01

**Authors:** Louis Costanzo, Bhesh R Karki, Brian Soto, Vladimir Falb, Cameron Page

**Affiliations:** 1 Internal Medicine, Downstate-Health Sciences University of New York, Brooklyn, USA

**Keywords:** thrombosis, pancreatitis, inferior vena cava, covid-19, congenital malformation, anticoagulation

## Abstract

An 86-year-old woman with a recent hospitalization for severe coronavirus disease 2019 (COVID-19) infection presented to the emergency department with abdominal discomfort and bilateral leg swelling. She was mildly tachycardic on physical exam, with superficial abdominal vessel dilation and bilateral lower extremity edema. Her laboratory results were significant for a mildly elevated lipase of 260 U/L (normal range: 0-160 U/L) and a positive COVID-19 PCR test. CT of the abdomen and pelvis did not show any pancreatic abnormality but revealed a duplicated inferior vena cava (IVC) with a thrombus located in the right IVC. The patient was subsequently placed on full-dose anticoagulation with the eventual achievement of clot lysis. It appears that the incidence of thrombosis, including IVC thrombosis, has been on the rise due to COVID-19-associated coagulopathy; therefore, a high index of clinical suspicion in these cases may prove to be lifesaving.

## Introduction

Congenital anomalies of the inferior vena cava (IVC) are extremely rare and have a reported incidence of 0.3-3% [1]. These anomalies, such as duplication of the IVC, are often found incidentally on advanced imaging and are usually of no clinical significance. Additionally, IVC thrombosis is an uncommon diagnosis; only 2.6-4% of patients with a lower extremity deep vein thrombosis (DVT) turn out to have an IVC thrombosis [2]. While an IVC thrombosis can present the clinician with a significant diagnostic and therapeutic challenge, a symptomatic IVC thrombosis in a person with a congenital malformation leading to duplicate IVCs may present an even greater challenge. 

We report a case of an elderly woman who presented with symptoms and signs of acute pancreatitis but was found to have a right inferior cava thrombosis on CTAP. This case report clearly highlights the symptoms associated with a duplicated inferior cava thrombosis and describes the management of this unique entity.

## Case presentation

An 86-year-old woman with a past medical history of insulin-dependent diabetes, hypertension, and hyperlipidemia was brought to the emergency department with a chief complaint of worsening abdominal pain over the past three weeks; she also had generalized weakness and bilateral lower extremity swelling. The abdominal pain was in the epigastric region, dull aching in nature, intermittent, non-radiating, and without any aggravating or alleviating factors. She had not taken any non-steroidal anti-inflammatory drugs (NSAIDs) recently and had no other complaints.

She had been recently hospitalized three weeks prior for coronavirus disease 2019 (COVID-19) infection complicated by pneumonia, which had been treated with remdesivir, dexamethasone, convalescent plasma, and prophylactic-dose low-molecular-weight heparin. During that hospitalization, a bilateral lower extremity ultrasound had been negative for DVT, and CT angiography of the chest had been significant for bilateral ground glass opacities, but showed no pulmonary embolism. She was discharged home with 3 liters of supplemental oxygen, but no additional anticoagulation. Her medications included amlodipine 5 mg daily, aspirin 81 mg daily, atorvastatin 20 mg daily, glimepiride 1 mg daily, and insulin glargine 14 units at night. Her last reported hemoglobin A1c was 7.5%; she had not undergone any recent surgeries, had no significant family history, was never a smoker, and did not consume alcohol.

On exam, she was normotensive but tachycardic to 105 beats per minute, afebrile, and without hypoxia or tachypnea. Her abdominal exam was significant for superficial vessel dilation, epigastric tenderness with deep palpation, and a negative Murphy’s sign. There were crackles heard anteriorly and posteriorly at the bases of the lungs and 2+ pitting bilateral lower extremity edema.

Laboratory testing was completed on admission with the following results - WBC count: 5.04 k/uL, hemoglobin: 11.8 g/dL, sodium level: 136 mmol/L, potassium level: 4.2 mmol/L, calcium level: 8.4 mg/dL, blood urea nitrogen (BUN): 15 mg/dL, creatinine: 0.9 mg/dL, aspartate aminotransferase (AST): 13 U/L, alanine aminotransferase (ALT): 14 U/L, alkaline phosphatase (ALP): 138 U/L, glucose: 280 mg/dL, lipase: 260 U/L (normal range: 0-160 U/L), lactate: 1.9 mmol/L, thyroid-stimulating hormone (TSH): 2.71 uIU/mL, and a urinalysis negative for red blood cells, white blood cells, or protein. A COVID-19 PCR test was performed, which eventually indicated a positive result. The D-dimer result from the previous hospitalization was >500 ng/mL (normal range: 220-500 ng/mL), but a new D-dimer level or additional inflammatory markers were not drawn on admission given the likelihood of elevation in the setting of a recent COVID-19 infection.

**Figure 1:**
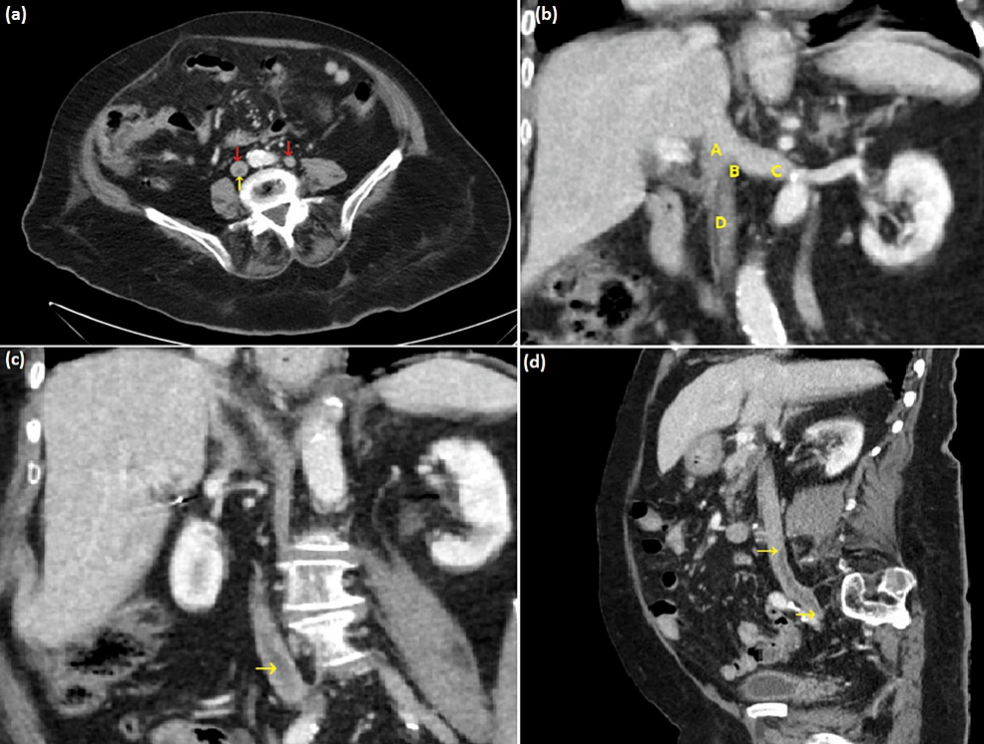
Duplication of the IVC (a) Axial CT image demonstrating a duplicated IVC (red arrows) with thrombus noted in the right IVC (yellow arrow); (b) Coronal view depicting (A) common IVC, (B) IVC confluence where both right and left IVC meet, (C) left IVC, and (D) right IVC; (c) Coronal view showing a central filling defect (yellow arrow) within the infrarenal portion of the right IVC; (d) Sagittal view demonstrating the extent of the thrombus (yellow arrows). It extends from the confluence of the right external and internal iliac veins to the infrarenal right IVC at the level of the L3 vertebral lower endplate CT: computed tomography; IVC: inferior vena cava

An ECG showed sinus tachycardia of 110 beats per minute with a normal rhythm. A bedside abdominal ultrasound did not reveal any pancreatic inflammation or gallstones. A CT of the abdomen and pelvis with intravenous contrast demonstrated a normal pancreas without peripancreatic fluid or stranding, colonic diverticulosis without diverticulitis, and a duplicated IVC with a thrombus noted within the right IVC (Figure 1), which extended from the confluence of right external and internal iliac veins to the infrarenal right IVC at the level of L3 vertebrae lower endplate.

Vascular surgery and interventional radiology were consulted. They discussed the options of catheter-directed thrombolysis and thrombectomy with the patient. However, a consensus was reached that no acute intervention would be undertaken given the duplicate IVC and increased risk of thrombus embolization if disturbed. The patient was already receiving prophylactic low-molecular-weight heparin of 40 mg/day, which was then appropriately increased to 1 mg/kg subcutaneously every 12 hours. A percutaneous transluminal angioplasty with stenting was also considered; however, the patient preferred pharmacological management rather than surgical intervention.

Intravenous fluids were administered at 120 mL per hour; the patient remained afebrile and was saturating well on room air. She was placed on airborne and contact precautions and monitored on continuous pulse oximetry. No additional treatment was started for COVID-19 and her newly confirmed positive test was attributed to viral shedding, which may last for longer than nine weeks [3]. In fact, the patient's first positive COVID-19 test had been documented approximately three weeks ago during her prior admission. The patient’s epigastric discomfort subsided during her hospital course and imaging did not show any signs of pancreatitis. While checking amylase or the isoenzyme of amylase may have favored a diagnosis of mild pancreatitis, these were not checked as the patient’s symptoms improved with treatment. Repeat lactate was found to be 1.1 after 24 hours of the presentation. A bilateral lower extremity ultrasound and chest CT were negative for additional clot formation.

The patient was continued on full-dose low-molecular-weight heparin for five days during her hospital course and was eventually discharged on apixaban 10 mg twice per day for seven days; she was instructed to take 5 mg twice daily for three months. At three months post-initiation of oral anticoagulants, she had complete resolution of symptoms, and apixaban was discontinued. She no longer had epigastric discomfort or dilated abdominal veins and her bilateral leg swelling improved.

## Discussion

On this patient's initial presentation, we anticipated diagnoses that were centered on epigastric pain. Acute pancreatitis was high on the differential given the clinical presentation, and we realized that mild pancreatitis could occur without abnormal CT findings [4]. But it was less likely because the pain was non-radiating to the back, there were no predisposing factors of pancreatitis, and we had an alternate diagnosis of IVC thrombus to better explain the discomfort. Although the patient lacked Murphy’s sign, gallbladder disease was still a possibility as this sign is less sensitive and specific in an elderly patient [5]. However, liver function tests were within normal limits and an ultrasound did not show any reflective echogenic focus within the gallbladder lumen. Next, we realized that an atypical presentation of myocardial ischemia was possible given her risk factors including advanced age, diabetes, hypertension, and hyperlipidemia. Other differentials including peptic ulcer disease, bowel obstruction, nephrolithiasis, or an infectious etiology were also considered. There was also the possibility that her abdominal pain was secondary to COVID-19, as over 20% of patients with COVID-19 present with GI-related complaints [6].

Anatomy of the IVC

The IVC is a vein with the largest diameter and it carries deoxygenated blood from the lower part of the body into the right atrium of the heart. It is formed by the union of the left and right common iliac veins at the fifth level of the lumbar vertebrae. Once formed, this large venous structure travels posterior to the abdominal cavity and runs along the right anterolateral side of the vertebral column. Because of this, the left-sided veins that empty into the IVC are typically longer than their counterparts. The IVC is a unique vessel, as it does not contain valves; instead, the forward flow of blood is driven by a pressure gradient created during respiration [7]. It ultimately passes through the tendinous region of the thoracic diaphragm at the level of T8, enters the thorax, and delivers blood to the right atrium below the superior vena cava. 

Many different veins empty directly into the IVC, including the hepatic and inferior phrenic veins at T8, the right suprarenal and renal veins at L1, the right gonadal vein at L2, the lumbar veins, and the right and left common iliac veins at L5. Clinical presentation often varies depending on the position of the IVC thrombus.

Duplication of the IVC during development

During the fourth to eighth week of embryological development, the IVC is derived from four different venous systems: the vitelline, subcardinal, supracardinal, and posterior cardinal. The majority of these veins regress, with the exception of the right supracardinal vein which forms the infrarenal IVC, and the distal portion of the posterior cardinal vein which forms the iliac confluence. Duplication of the IVC occurs when the left supracardinal vein fails to regress, thereby leaving both the right and left supracardinal veins to persist [8]. This anomaly is ordinarily of no clinical significance and not generally detected until a patient undergoes advanced abdominal imaging for an unconnected purpose.

An IVC thrombus mimics the pathophysiology of DVT [9]. Virchow’s triad, named after clinician and researcher Rudolf Virchow who worked on thrombosis in the 1800s, describes three categories that contribute to venous clot formation. These are stasis or turbulence, endothelial injury, and hypercoagulability [10]. This triad can aid in the explanation of the pathophysiology leading to an IVC thrombosis. In a patient with an IVC without any congenital abnormalities, thrombosis is often associated with compression by tumors, abscesses, masses, or aneurysms. These compressive forces result in pressure changes that cause stasis of blood; fulfilling one of the criteria established by Virchow’s triad. However, these are not the only etiologies, as patients who are born with conditions that predispose them to hypercoagulable states [11] are at risk for any venous thrombosis. Trauma, such as the insertion of dialysis or femoral catheters, or occlusion of IVC filters, also plays a role in IVC thrombosis due to vessel wall damage [12].

In patients with congenital IVC abnormalities, as in our patient, the pathophysiology of thrombus formation likely begins with the turbulent flow of blood through two separate IVC vessels [13]. This turbulent blood flow within this large vessel not only leads to endothelial damage but also forms countercurrent pockets of venous stasis, thus promoting thrombus formation. We believe that the recent COVID-19 infection in our patient added an extra insult as hypercoagulability in addition to the turbulent blood flow. COVID-19 has been described as a hypercoagulable state and is frequently associated with thrombus formation [14].

IVC thrombosis and COVID-19

IVC thrombosis represents a subset of DVT, and hence Virchow’s triad of stasis, endothelial injury, and hypercoagulability continues to be the foundation for our understanding of the mechanism of thrombosis. As previously mentioned, in patients with congenital abnormalities of the IVC, thrombosis may be due to turbulent blood flow through two large vessels and the creation of pockets of venous stasis; or it may occur in the presence of a hypercoagulable state such as COVID-19 infection.

**Figure 2:**
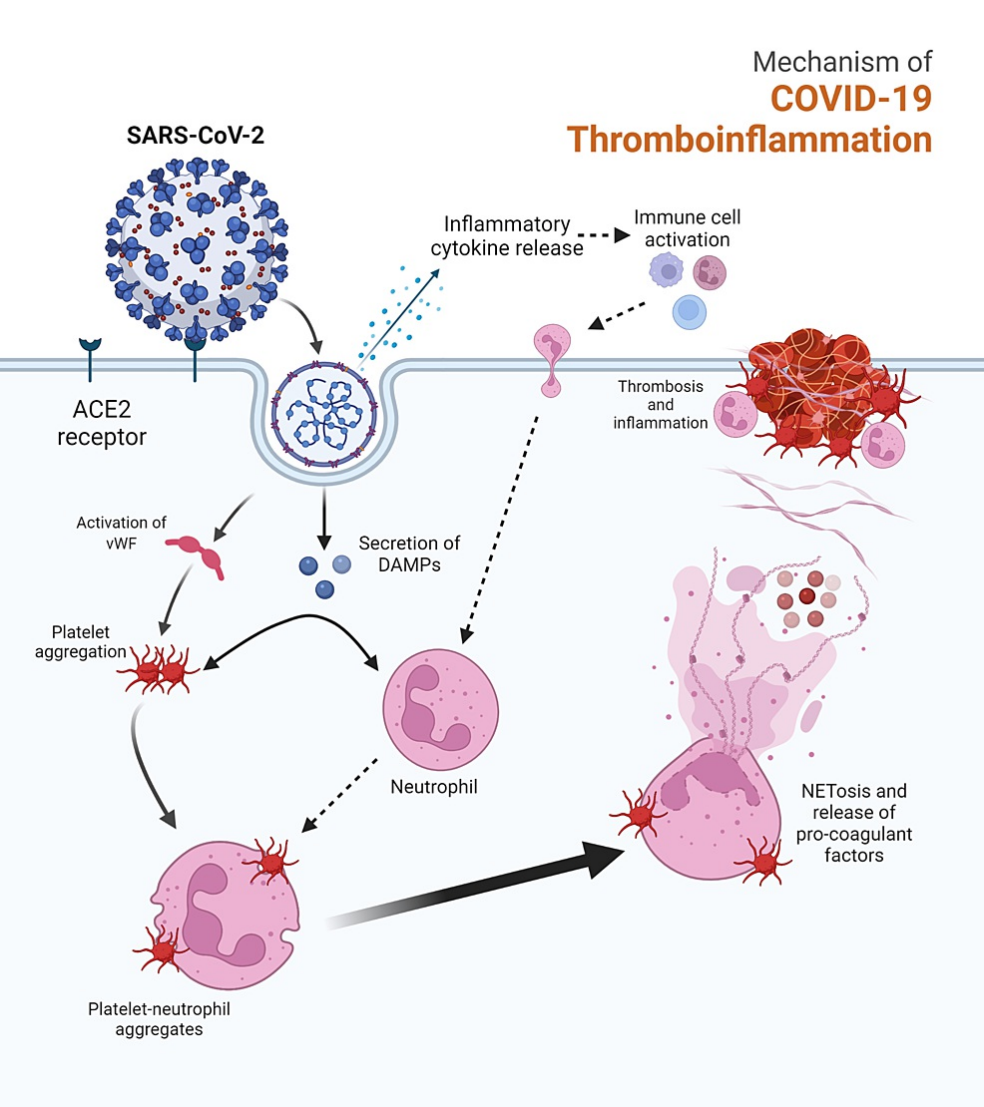
Mechanism of COVID-19 thromboinflammation SARS-CoV-2 spike protein binds to ACE-2 receptors, facilitating entry into the cell. This stimulates the release of damage-associated molecular patterns (DAMPs) and the release of cytokines/chemokines which recruit immune cells. Viral entry also leads to cell damage causing von Willebrand factor release, platelet activation, leukocyte-platelet aggregation, and NETosis, all of which contribute to thrombosis and further inflammation ACE-2: angiotensin converting enzyme-2; COVID-19: coronavirus disease 2019; SARS-CoV-2: severe acute respiratory syndrome coronavirus 2 Image created with BioRender.com

COVID-19 has been implicated in various coagulation abnormalities and microcirculatory thrombosis. This complex process involves endothelial cell death, the release of damage-associated molecular patterns (DAMPs), pro-inflammatory cytokine release, immune cell activation, activation of both the coagulation and complement pathways [15], and NETosis [16] (Figure 2). Given our patient’s recent hospitalization for a severe COVID-19 infection, we considered this as a possible etiology of clot formation.

Severe acute respiratory syndrome coronavirus 2 (SARS-CoV-2) binds to ACE-2 receptors on endothelial cells, which facilitates viral entry into the cell leading to endothelial destruction. Given the endothelial injury, significant amounts of von Willebrand factor (vWF) are secreted and platelets are activated. This may lead to the activation of the coagulation cascade [17]. Inflamed tissue promotes chemokine and cytokine release, which in turn commands multiple cell populations including neutrophils, macrophages, dendritic cells, and natural killer cells to the site of infection. Platelets form platelet-leukocyte aggregates, which have been implicated in the pathophysiology of COVID-19 [18] and thrombosis formation [19]. These aggregates stimulate the release of neutrophil extracellular traps (NETs) [20], which are web-like chromatin fibers, DNA, and histones that promote vessel occlusion [21]. While it is difficult to determine if our patient’s symptomatic right IVC thrombosis was due to a previous severe COVID-19 infection, this mechanism may have contributed to the formation of the IVC clot.

Symptoms and workup of IVC thrombosis

**Figure 3:**
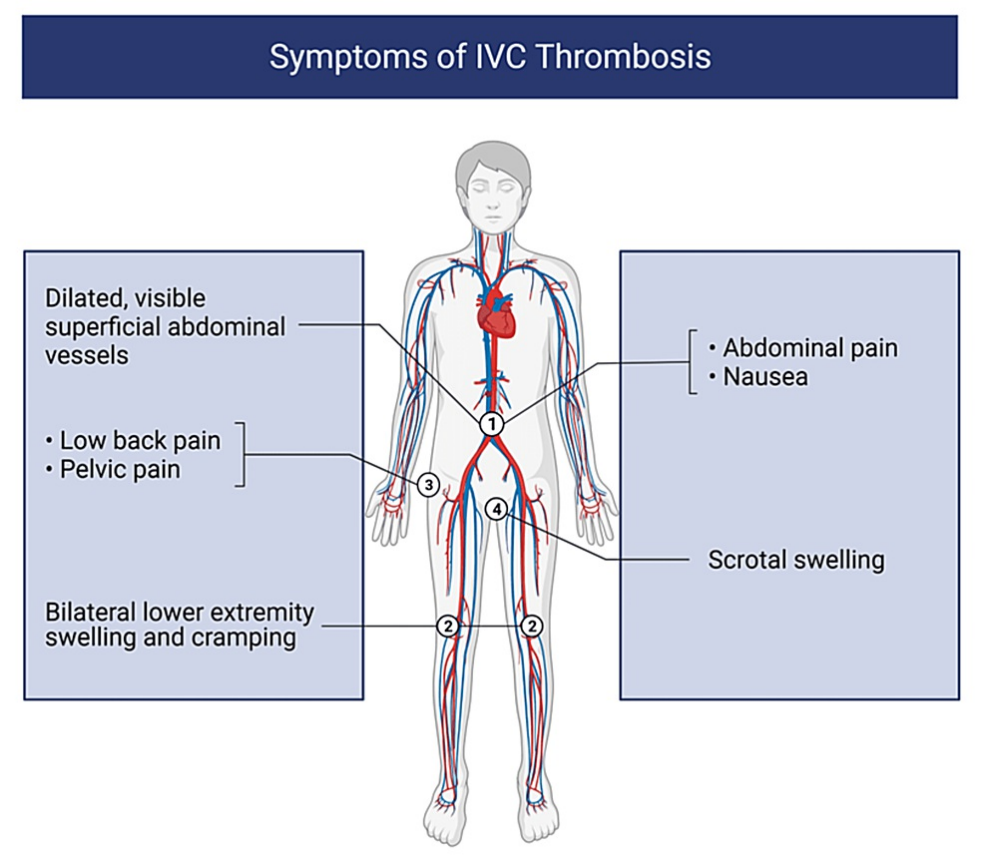
Symptoms of IVC thrombosis IVC thrombosis and duplicate IVC thrombosis symptoms are similar in nature. Dilated superficial abdominal veins (1) and bilateral lower extremity swelling (2) are commonly seen. Low back pain (3) and scrotal swelling in men (4) may also be observed IVC: inferior vena cava Image created with BioRender.com

The symptoms associated with IVC thrombosis (Figure 3) are directly related to the level of clot formation; nearly 50% of patients will present with dilated superficial abdominal veins (which provide collateral circulation when the IVC is obstructed) and bilateral lower extremity swelling (due to an increase in venous blood pressure) [22]. Other symptoms encountered include abdominal pain or cramping (as seen in our patient), lower back or pelvic pain, signs of hemodynamic instability, and scrotal swelling in men [23].

Because this condition is somewhat unusual and not regularly considered as part of a differential diagnosis, early laboratory testing may not be of much significance. However, suspicion should be raised if the patient has increased inflammatory markers, abnormal coagulation factors, and a high D-dimer level. Elevated C-reactive protein and D-dimer levels were reported in 10 published cases of IVC thrombosis, all of which were caused by congenital malformation [24]. However, it is important to consider that these markers may be elevated in the setting of a COVID-19 infection.

Our patient likely had a mildly elevated lipase without overt signs of pancreatitis or malignancy. Congestion of the IVC system can lead to mild pancreatic damage not observed on imaging, though venous congestion occurred below the level of the thrombus in our patient, which may not contribute to the elevated lipase. However, it should be mentioned that hyperlipasemia has been noted in a small subset of patients with COVID-19, though this has reportedly been observed in those with significant gastrointestinal manifestations [25].

Though there are no guidelines to aid in the diagnosis of IVC thrombosis, duplex ultrasound scan (USS), computed axial tomography (CT/CAT), MRI, and direct catheter venography are useful. While the USS is noninvasive and may be used first, potential limitations of this modality include difficulty in applying this in patients with morbid obesity and obstructing views due to bowel gas. Thrombosis can be identified on a USS if the IVC waveform appears monophasic [26] with high velocities, as opposed to displaying a continuous waveform with a respiratory variation. However, if there is a concern for a thrombus on USS, a CT scan can accurately diagnose the location and extent of the IVC thrombus. Though MRI is the most accurate modality, it is costly and usually not readily accessible. Direct catheter venography, though not commonly used, may allow for an absolute diagnosis. 

Treatment of IVC thrombus

It is important to consider patient-specific factors, timing, and associated conditions when deciding on treatment options for IVC thrombosis. The ultimate goal is to minimize the risk of a pulmonary embolism and alleviate symptoms. If the thrombus is idiopathic, that is, without any associated causes, the treatment involves anticoagulation, but thrombectomy is regarded as the safest option [11]. The use of unfractionated heparin, or low-molecular-weight heparin, can reduce the risk of embolization at the time of diagnosis [27]. Unfortunately, clot lysis is seldom achieved in a timely fashion and systemic thrombolysis with streptokinase or tissue plasminogen activator may be more effective. Wilson et al. have reported a case of an IVC thrombus treated successfully with low-dose infusional streptokinase [28]. However, there is a significant risk of bleeding when using systemic-based thrombolytic medications.

Catheter-directed thrombolysis (CDT), a minimally invasive procedure, is beneficial if administered within 14 days in symptomatic patients with an IVC thrombosis [29]. There is currently no consensus regarding the benefits of placing an IVC filter to minimize the risk of thrombus migration in these patients. However, angioplasty and stenting can be considered for patency. Hartung et al. reported a 98% success rate when managing ilio-caval obstructive lesions in 89 patients with endovenous angioplasty with stenting [30]. These are minimally invasive techniques that provide good long-term patency rates.

Vena caval anomalies are risk factors for venous stasis and recurrent thrombosis; however, there is no current consensus on treatment duration. When considering initial anticoagulation therapy for non-high-risk venous thromboembolism, the first-line therapy includes direct oral anticoagulants such as apixaban, rivaroxaban, dabigatran, or edoxaban [31], which should be given for a minimum of three months. For those patients with first-episode idiopathic venous thrombosis, oral anticoagulation can be considered for more than six months; however, Agnelli et al. have determined that the benefit associated with extending the duration of anticoagulant therapy to one year is not maintained after the therapy is discontinued [32]. Therefore, it does not seem unreasonable to provide an indefinite course of oral anticoagulation in those with venous anomalies and an IVC thrombosis, given their increased risk of recurrent thrombosis. However, it is important to consider the age of the patient and symptomatology when considering a prolonged course of anticoagulation. Given our patient's age and resolution of symptoms at three months, we stopped oral therapy at that time.

## Conclusions

Duplication of IVC is a rare congenital condition that is usually of no clinical significance. The anomaly involves a failure of left supracardinal regression and is often incidentally found on advanced imaging. Clinicians should suspect an IVC thrombosis in patients presenting with both dilated superficial abdominal veins and bilateral lower extremity edema. Patients may also present with nonspecific symptoms such as abdominal pain, back pain, or scrotal swelling in males. The incidence of thrombosis, including those of the IVC, has likely increased due to the hypercoagulability state of COVID-19; therefore, clinicians should have a high level of suspicion when evaluating COVID-19-positive patients with abdominal findings and lower extremity swelling.

The most common imaging modalities to support the diagnosis include a duplex USS, computed axial tomography, MRI, and direct catheter venography. Once a diagnosis is made, it is important to initiate anticoagulation to reduce embolization, but this rarely leads to clot lysis in a timely fashion. The favored treatment modalities are thrombectomy and endovascular interventions such as percutaneous balloon angioplasty and stenting.
